# Epidemiology, haematology and molecular characterization of haemoprotozoon and rickettsial organisms causing infections in cattle of Jammu region, North India

**DOI:** 10.1186/s12917-021-02915-9

**Published:** 2021-06-15

**Authors:** Rabjot Kaur, Anish Yadav, Shafiya I. Rafiqi, Rajesh Godara, Vikrant Sudan, D. Chakraborty, Rajesh Katoch

**Affiliations:** 1 Department of Veterinary Parasitology, Khalsa college of Veterinary and Animal Sciences, Amritsar, Punjab 143001 India; 2 Division of Veterinary Parasitology, Faculty of Veterinary Sciences and Animal Husbandry, SK University of Agricultural Sciences and Technology of Jammu. RS Pura, Jammu, J&K 181102 India; 3 Department of Veterinary Parasitology, U P Pandit Deen Dayal Upadhyaya Pashu Chikitsa Vigyan Vishwavidyalaya Evam Go Anusandhan Sansthan (DUVASU), Mathura, 281001 India; 4 Division of Animal Genetics and Breeding, Faculty of Veterinary Sciences and Animal Husbandry, SK University of Agricultural Sciences and Technology of Jammu. RS Pura, Jammu, J&K 181102 India

**Keywords:** *Anaplasma*, *Babesia*, Cattle, Haematology, Molecular characterisation, *Theileria*

## Abstract

**Background:**

The present study was aimed at establishing the prevalence, epidemiology and molecular characterization of major haemoprotozoons (*Babesia* and *Theileria*) and rickettsia (*Anaplasma*) of cattle in Jammu region (North India) using microscopy and Polymerase Chain Reaction (PCR). Hematology, microscopy and PCR based prevalence studies were undertaken with 278 whole blood samples from cattle. Molecular prevalence studies were followed by genetic characterization of the isolates of *Babesia*, *Anaplasma* and *Theileria* spp. based on 18S rRNA, 16S rRNA and Tams1 gene, respectively. The data related to metrology and epidemiological variables like temperature, rainfall, season, age and type of livestock rearing was analyzed and correlated with occurrence of disease by statistical methods.

**Results:**

The prevalence based on microscopy was 12.9% (36/278) whereas PCR recorded 30.22% (84/278) animals positive for haemoparasitic infections. All the samples found positive by microscopy were also recorded positive by PCR. Thus the study revealed prevalence of *Babesia bigemina*, *Anaplasma marginale* and *Theileria annulata* to be 9.7, 16.5 and 0.7% respectively. The metrological and epidemiological variables made inroads for the propagation of vector ticks and occurrence of infection. Haematological alterations predominantly related to decrease in haemoglobin, red blood cell count and packed cell volume were evident in diseased animals and collaterally affected the productivity. Further the genetic characterization of *Babesia bigemina*. (MN566925.1, MN567603, MN566924.1), *Anaplasma marginale*. (MH733242.1, MN567602.1) and *Theileria annulata* (MT113479) provided a representative data of the isolates circulating in the region and their proximity with available sequences across the world.

**Conclusions:**

Despite holding much significance to the animal sector, comprehensive disease mapping has yet not been undertaken in several parts of India. The present study provides a blue print of disease mapping, epidemiological correlations and genomic diversity of *Babesia bigemina*, *Anaplasma marginale* and *Theileria annulata* circulating in the region.

**Supplementary Information:**

The online version contains supplementary material available at 10.1186/s12917-021-02915-9.

## Background

India is primarily an agricultural country and the losses incurred as a result of tick borne diseases (TBDs) to livestock in India are huge, estimated to be 8.7 million USD, predominantly affecting the small and marginal farmers. Among the various tick transmitted diseases babesiosis, theileriosis, anaplasmosis and ehrlichiosis are the significant ones that drive attention. Babesiosis is considered as economically important tick-borne haemoprotozoan disease in tropical and subtropical countries, the most prevalent species being *Babesia bovis* and *Babesia bigemina* [1]. *Babesia bigemina,* transmitted by brevirostrate tick, *Rhipicephalus (Boophilus microplus)* is usually associated with high parasitemia. Anaplasmosis is a rickettsial disease transmitted biologically through ticks mainly by *Rhipicephalus (Boophilus) microplus* [2]. It is endemic in some parts of the world [3, 4] while in India incidence of *A. marginale* infection has been recorded in livestock [5–7]. The *Theileria* species that infect bovines in India are *T. annulata* and *T. orientalis*, and both species are transmitted by *Hyalomma anatolicum*. The most common clinical signs observed in TBDs are fever, inappetence and mortality, with peculiar hemoglobinuria and anemia for bovine babesiosis [4], and enlarged lymph nodes for theileriosis.

Conventional microscopy is often used for the diagnosis of these diseases [5] but, cryptic cases and carrier states are often misdiagnosed or not diagnosed at all, besides low sensitivity and lack of species level differentiation [8]. Similarly, sero-diagnosis suffers from the problem of cross-reactivity and sometimes low antigen output. Microscopy along with sensitive and specific molecular tools like PCR may help in mapping the diseases and various risk factors for the transmission of major TBDs of livestock viz. babesiosis, theileriosis and anaplasmosis. In the light of the fact that 39 million crossbred cattle of India, meant to boost productivity, are at potential risk of contracting TBDs; it becomes imperative to model the prevalence, identify the risk factors and devise control strategies for the control of tick and TBDs of livestock.

The present study was aimed to generate baseline data about the prevalence of tick borne haemoprotozoan and rickettsial infections in cattle in Jammu province of North India, based on conventional and molecular techniques, assess the risk factors and haematological alterations associated with the disease. Moreover, phylogenetic relatedness and genetic diversity of the isolates infecting cattle was also investigated to aid in epidemiological surveillance.

## Results

### Prevalence studies based on microscopy and PCR

**Fig. 1:**
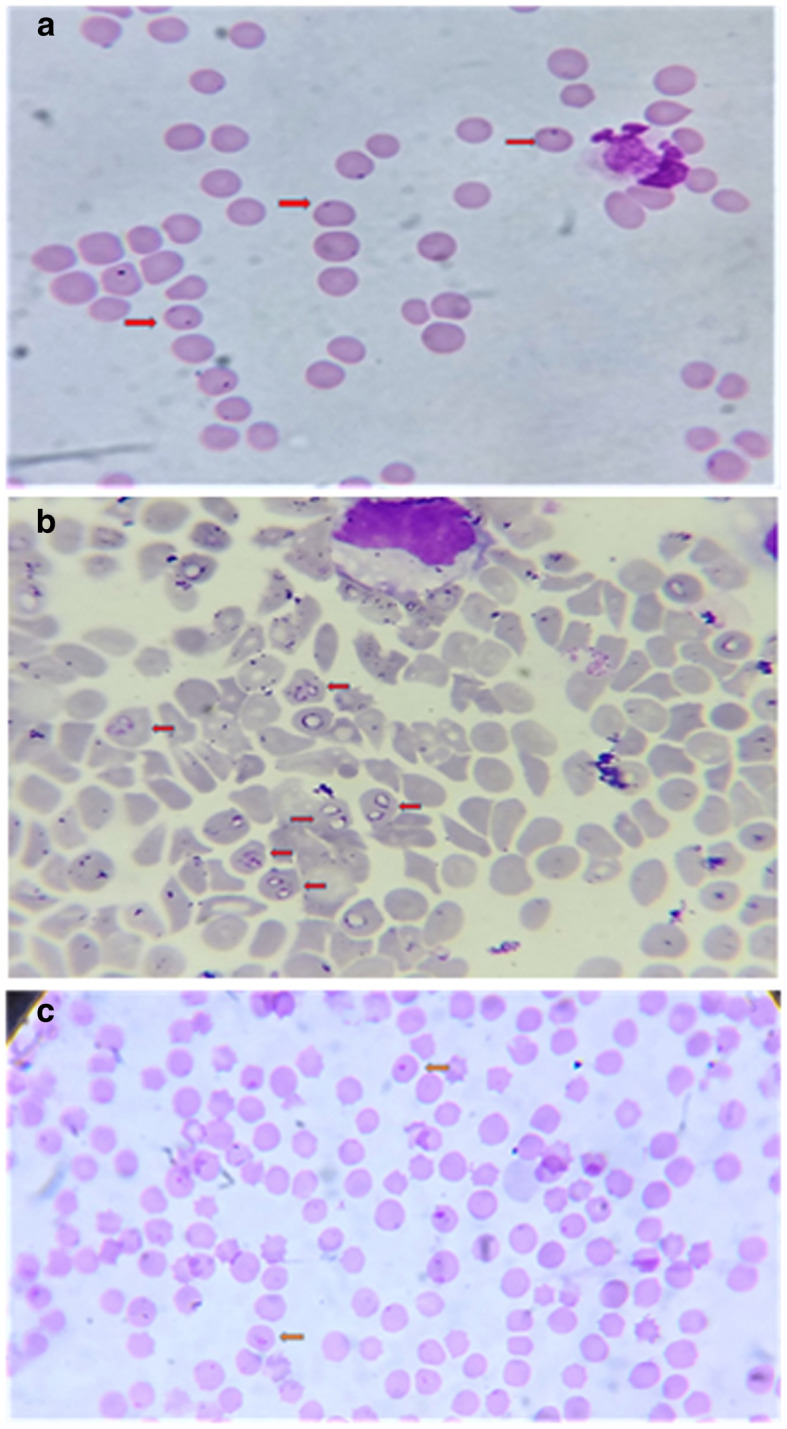
**a** Blood smear positive for *Anaplasma spp* (1000x). **b** Blood smear positive for *Babesia* piroplasms (1000x). **1c** Blood smear positive for *Theileria* piroplasms (1000x)

All the samples found positive by microscopy were also recorded positive by PCR. Microscopy revealed infection in 12.94% animals (36/278) (Fig. 1a, b, c) whereas 30.21% samples (84/278) were diagnosed as positive through PCR assay. The PCR assay could effectively diagnose cryptic and sub clinical cases and was found significantly sensitive (*χ*2 23.475, odds ratio, OR = 2.911, 95% CI = 1.886–4.491) as compared to microscopy. The prevalence of *A. marginale* was (46/278) 16.5% followed by *Babesia bigemina* (27/278, 9.7%) and *T. annulata* (2/278, 0.7%). Concurrent haemoprotozoan and rickettsial infections were detected in 9 (3.2%) animals (Table 1).

**Table 1:** Prevalence of haemoparasites by microscopy and PCR

	Positive by Giemsa staining of thin blood smear			Positive by PCR		
	Sample Size	Positive (%)	95% CI	Sample Size	Positive (%)	95% CI
***Babesia***	278	12 (4.3)	2.32–6.32	278	27 (9.7)	1.78–2.91
***Anaplasma***	278	20 (7.1)	4.65–9.74	278	46 (16.5)	2.23–3.66
***Theileria***	278	1 (0.3)	−0.23 - 0.95	278	2 (0.7)	0.51–0.83
**Mixed infection**	278	3 (1.0)	0.06–2.10	278	9 (3.2)	1.06–1.74
**Total**	278	36 (12.9)	9.65–16.25	278	84 (30.2)	2.75–4.52

### Attribution of clinical signs to the disease

The main clinical signs exhibited were pyrexia, decreased milk production, pale mucous membrane, tick infestation and anorexia. Haemoglobinuria was observed in babesiosis and lymph node enlargement manifested in theileriosis. Tick infestation was recorded in 64/84 (76.19%) of animals positive for haemoparasites. Decreased milk production was found in 72/84 (85.71%) animals. Among 31 animals showing symptoms of pica, 30 were found positive for anaplasmosis.

### Assessment of risk and impact of environmental variables

**Fig. 2:**
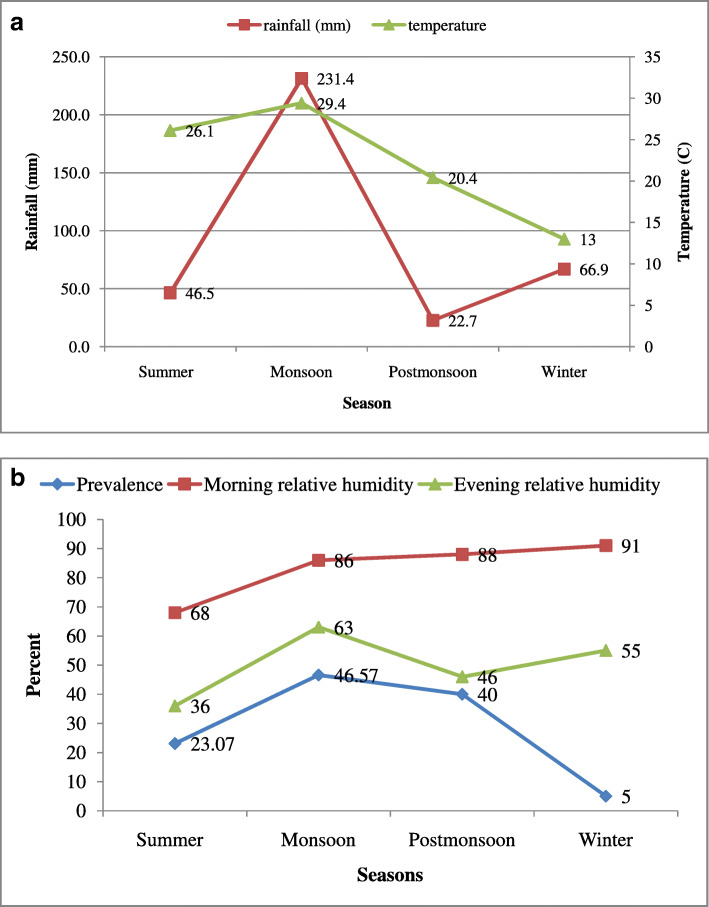
**a**: Graph depicting rainfall (in mm) and temperature (°C) during different seasons of the year in Jammu region. **b:** Graphical representation of morning and evening relative humidity and prevalence during different seasons of the year in Jammu region

Evaluation of risk factors for occurrence of disease in the present study was undertaken by modelling effect of age, season, breed, sex and type of farm. A significant effect of season and host age on occurrence of diseases was indicated. The highest prevalence of haemoprotozoan and rickettsial infection was found in animals having > 3 years of age (45/120, 37.5%) followed by 1–3 years (30/105, 28.5%) and < 1 year of age (9/53, 16.9%). The overall prevalence did not vary significantly (*p* = 0.629) between male and female animals. Unorganised farms without proper flooring, cracked walls and poor sanitation showed significantly (*p* = 0.007) higher prevalence of infection (60/160, 37.5%,) as compared to organised farms (24/118, 20.3%, Cross bred animals revealed higher infection (33.6%) compared to indigenous animals (15.3%), reiterating the fact that cross bred animals are at higher risk than native breeds. The probability of occurrence of infections in monsoon was 16.56 times higher than winter. The correlation of environmental variables with the prevalence presented a clear picture of epidemiological interventions in the disease occurrence (Fig. 2 a and b, supplementary data 1). The detailed table of risk factors associated with prevalence of haemoprotozoon and rickettsial infections among cattle is presented in Table 2.

**Table 2:** Risk factors associated with prevalence of haemoparasites among cattle in Jammu, North India according to different variables

	Variable	Samples	No of animals positive (% positive for pathogen)				Total animals Positive (%)	95% CI	Chi Square	df	***P*** value	Odds ratio	95% CI
			***Anaplasma marginale***	***Babesia bigemina***	***Theileria annulata***	Mixed							
**Farm Management**	Organized	118	11 (9.3)	10 (8.4)	0	3(2.5)	24 (20.3)	14.2–26.4	9.49	1	(*P* < 0.01)	constant	–
	Unorganized	160	35 (21.8)	17(10.6)	02 (1.2)	6(3.7)	60(37.5)	31.2–43.7				2.35	1.3–4.0
**Age**	< 1 year	53	6(11.3)	2(3.7)	0	1(1.8)	9(16.9)	8.5–25.4	7.56	2	(*P* < 0.05)	constant	–
	1–3 year	105	15(14.2)	11(10.4)	1(0.9)	3(2.8)	30(28.5)	21.3–35.8				0.84	0.3–2.1
	> 3 year	120	25(20.8)	14(20.8)	1(0.8)	5(4.1)	45(37.5)	30.2–44.7				2.93	1.3–6.5
**Season**	Winter	60	2(3.3)	1(1.6)	0	0	3(5.0)	0.3–9.6	32.56	3	(P < 0.01)	constant	–
	Summer	65	8(12.3)	6(9.2)	0	1(1.5)	15(23.0)	14.5–31.6				5.7	1.5–20.8
	Monsoon	73	20(27.3)	9(12.3)	1(1.3)	4(5.4)	34(46.5)	37.0–56.1				16.56	4.7–57.7
	Post monsoon	80	16(20.0)	11(13.7)	1(1.2)	4(5.0)	32(40.0)	31.0–48.9				12.6	3.6–43.9
**Breed**	Indigenous	52	5(9.6)	3(5.7)	0	0	8(15.3)	7.1–23.5	6.67	1	(P < 0.01)	constant	–
	Cross bred	226	41(18.1)	24(10.6)	2(0.8)	9(3.9)	76(33.6)	28.4–38.7				2.79	1.2–6.2
**Sex**	Male	12	1(8.3)	1(8.3)	0	0	2(16.6)	−0.9 – 34.3	1.09	1	(*P* > 0.05)	constant	–
	Female	266	45(16.9)	26(9.7)	2(0.7)	9(3.3)	82(30.8)	26.1–35.4				2.29	0.4–10.4

### Haematological alterations

Haematological findings of infected animals (*n* = 84) were compared with healthy control (*n* = 10) animals. A significant decrease in the Hb (*p* = 0.013), PCV (*p* = 0.001) and RBC (*p* = 0.027) count of animals with babesiosis was recorded (Table 3). The RBC count was also found significantly decreased in animals harbouring mixed infection.

**Table 3:** Haematological alterations in animals with haemoprotozoon and rickettsial infection

Haematological Parameter	Non infected control (*n* = 10)	*Babesia* spp.(*n* = 27)	*Anaplasma* spp.(*n* = 46)	*Theileria* spp.(*n* = 2)	Mixed infection (*n* = 9)
Hb (g/dl)	10.65 ± 1.02^b^	6.16 ± 0.48^a^	7.41 ± 0.55^ab^	7.08 ± 2.07^ab^	7.22 ± 1.09^ab^
PCV (%)	28.79 ± 1.32^c^	17.67 ± 1.39^a^	22.91 ± 1.26^bc^	19.50 ± 4.5^ab^	18.22 ± 2.22^ab^
RBC (10^6^/mm^3^)	5.82 ± 0.56^b^	3.07 ± 0.22^a^	4.27 ± 0.43^ab^	3.40 ± 0.70^ab^	3.28 ± 0.84^a^
WBC (×10^3^/ μl)	10.52 ± 0.96^ab^	6.39 ± 0.31^a^	11.98 ± 1.07^b^	9.00 ± 2.0^ab^	9.83 ± 1.41^ab^
LYM (%)	62.96 ± 3.13	59.93 ± 2.76	60.24 ± 2.26	73.50 ± 7.5	59.89 ± 4.40
MON (%)	1.54 ± 0.1	1.55 ± 0.21	1.56 ± 0.14	1.00 ± 0.5	1.60 ± 0.36
GRA (%)	36.77 ± 1.54	40.62 ± 2.12	40.19 ± 1.3	27.50 ± 6.5	39.67 ± 3.46
MCH (pg)	15.82 ± 1.30	14.54 ± 1.25	14.63 ± 1.01	14.50 ± 3.5	13.22 ± 2.22
MCHC (g/dl)	34.62 ± 1.23^a^	45.69 ± 2.54^b^	32.02 ± 1.0^a^	36.50 ± 7.5^ab^	34.67 ± 2.62^a^
MCV (fl)	46.42 ± 1.97	47.54 ± 2.47	48.21 ± 1.21	51.50 ± 5.50	45.11 ± 3.35

Values of Hb (g/dl), PCV (%), RBC (10^6^/mm3), WBC (×10^3^/ μl), and MCHC (g/dl) with different superscripts a,b,c differ significantly in a row. *Hb* Haemoglobin; *PCV* Packed cell volume; *RBC* Red blood cells; *WBC* White blood cells; *LYM* Lymphocytes; *MON* Monocytes; *GRA* Granulocytes; *MCH* Mean corpuscular haemoglobin; *MCHC* Mean corpuscular haemoglobin concentration; *MCV* Mean corpuscular volume

### Polymerase chain reaction and phylogenetic analysis

**Fig. 3:**
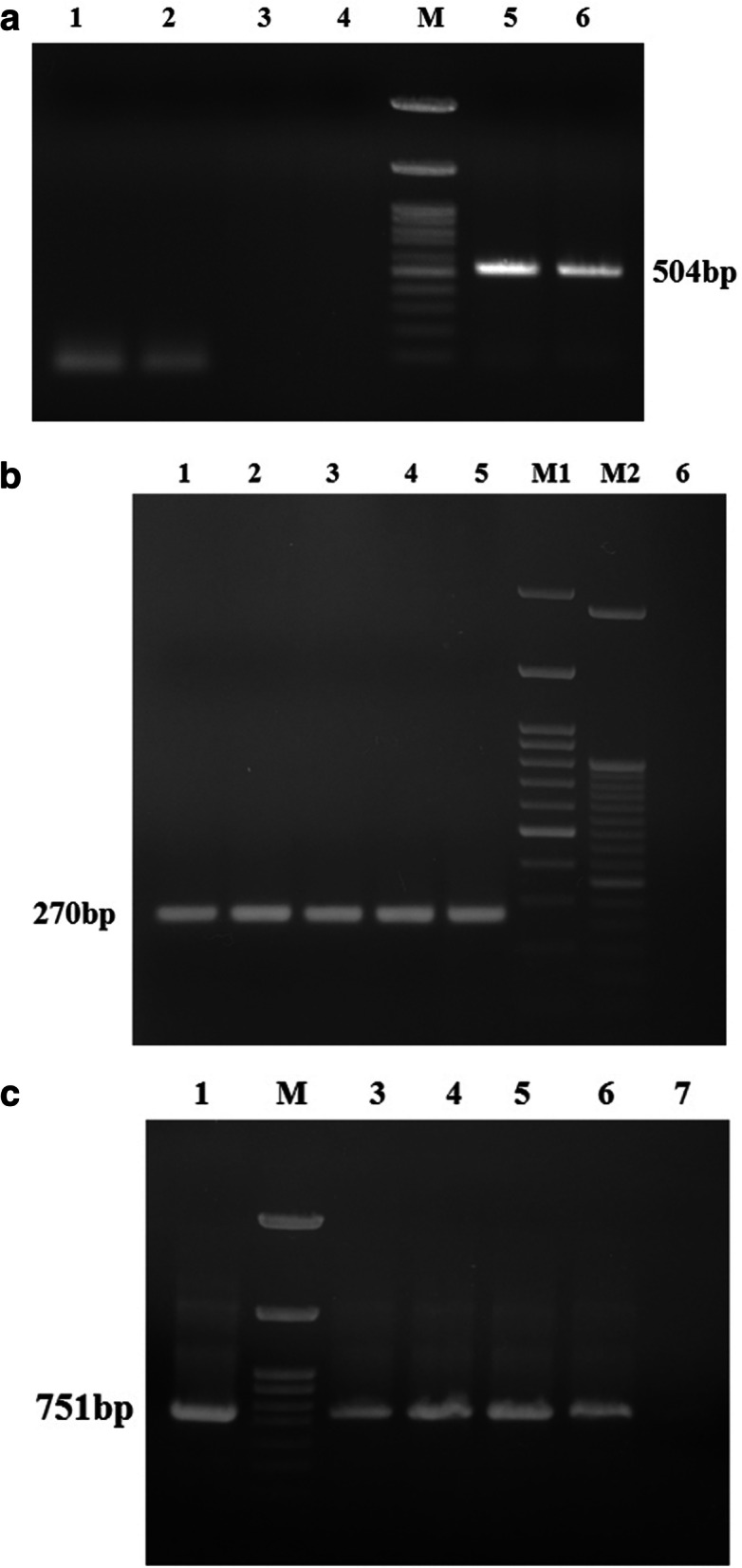
**a**: PCR amplification of 504 bp *Babesia spp.* 18S rRNA, Lane 1, 2 negative control, Lane 3, 4, 5: test samples M: 100 bp ladder, Lane 6: positive control. **b** PCR amplification of 270 bp *Anaplasma marginale* 16 s rRNA, Lane 1–4 test samples, M1: 100 bp ladder, M2: 50 bp ladder, Lane 5: positive control, Lane 6 negative control. **c** PCR amplification of 751 bp *Theileria annulata* Tams1 gene, Lane 1: positive control, M: 100 bp ladder, Lane 3,4,5,6,: test samples at gradient, Lane 7: negative control

In the PCR assays, specific amplifications of 504 bp, 270 bp and 751 bp products were obtained for 18S rRNA gene of *Babesia*, 16S rRNA gene of *A. marginale* and Tams1 gene of *T.annulata* (Fig.3 a, b and c). Six PCR products comprising of three *B. bigemina*, two *A. marginale* and one *T. annulata*, representing different isolates of Jammu region were sequenced. The sequence similarity searches in BLAST revealed that Jammu isolate (MN566925.1) of *Babesia* spp. was 99.8% identical to Kathua (MN567603), Udhampur (MN566924.1) and Meghalaya (KF606864.1) isolates of *Babesia bigemina* and had marked similarity with *B. bigemina* from Argentina (HQ688688.1) and USA (MH050387.1). At position 363 C was replaced with G in Udhampur and Kathua isolates (*Babesia bigemina* alignment, supplementary data 2). Two sequences of *A. marginale* from our study, submitted to GenBank (MH733242.1, MN567602.1), revealed 100% nucleotide identity with published sequences of *A. marginale* of French (MN317256.1), Iranian (MK310488.1) and Vietnamese (MH686047.1) origin. The only sequence of *T.annulata* from Jammu region (MT113479) was found to be 99% similar to Indian isolates from Izatnagar (MF346013.1) and Hissar (AF214840.1). In Jammu isolate at position 137 C was replaced by A, A by G (607) and C by A (611) in comparison to Izatnagar isolate (*T.annulata* alignment, supplementary data 3). Moreover there were 6 nucleotide substitutions in Jammu isolate vis-à-vis Hisar isolate at position 365 (C to A), 368(C to A), 389 (A to G), 399 (A to C), 414 (G to C) and 425 (T to C). Furthermore, the Jammu isolate showed 99.07% sequence homology to United Kingdom W1_2 isolate (KX981026.1).

**Fig. 4:**
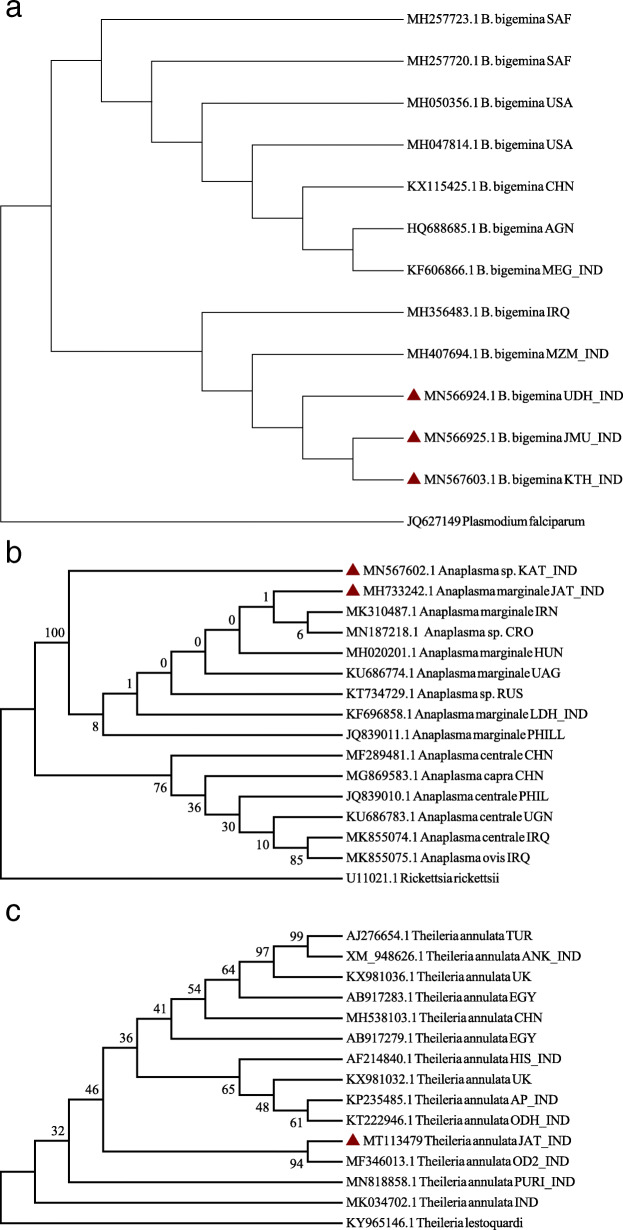
**a** Phylogenetic relationship of *B. bigemina* isolates based on 18S rRNA gene. All accession numbers correspond to different *B. bigemina* isolates followed by their country of origin. The sequences generated in the present study are marked as red triangle. **b** Phylogenetic relationship of *A. marginale* isolates based on 16S rRNA gene. All accession numbers corresponds to different *Anaplasma* isolates followed by their country of origin. The sequences generated in the present study are marked as red triangle. **c**. Phylogenetic relationship of *T. annulata* isolates based on Tams1gene. All accession numbers corresponds to different *T. annulata* isolates followed by their country of origin. The sequence generated in the present study is marked as red triangle

In the phylogenetic tree, our sequences clubbed together with Mizoram isolate (MH407694.1) forming separate Indian subclade (Fig. 4a). The phylogenetic tree of *A. marginale* (Fig. 4b) showed species level diversity in which Kathua isolate branched away from Ludhiana isolate (India, KF696858.1). The only *T. annulata* sequence from the present study formed a separate clade with Indian isolate of *T. annulata* (MF346013.1) on the phylogenetic tree (Fig. 4c).

## Discussion

In developing countries like India considerable economic losses occurs in large and small holding livestock productivity farming system due to TBDs [6]. Prevalence studies are of immense importance for disease mapping and investigating the epidemiological triad. The agro-climatic conditions of the Jammu region are highly favourable for growth and multiplication of ticks which act as natural vectors of theileriosis, babesiosis and anaplasmosis. In the present study, high prevalence of *Babesia bigemina* (14.02%, 39/278), *A. marginale* (23.74%, 66/278) and low prevalence of *Theileria annulata* (1.079%, 3/278) can be attributed to *Rhipicephalus* (*Boophilus*) *microplus* being the only tick which infests the bovines of Jammu region [12]. A thorough review of literature reveals varying incidence of babesiosis ranging from 0.76 to 18.50% in India [13–15]. *Anaplasma* is also associated with a long term/life long carrier state [16] and so the probability of detecting positive animals, particularly in areas of endemic instability, is increased, primarily with use of molecular tools [6, 17]. The animals found positive for theileriosis had a history of importation from the neighbouring state, Punjab, having high prevalence of *H. anatolicum* [18] and *T. annulata* [5]. Owing to meagre presence of vector tick (*H. anatolicum)*, considerably low prevalence of theileriosis was found in union territory of Jammu and Kashmir [19]. Livestock owners import high yielding animals from bordering states of Punjab and Haryana without proper quarantine and thus carrier cattle get transported to non endemic areas, increasing the likelihood of transboundary outbreaks. PCR assay has been employed for sensitive detection of haemoparasites in many diagnostic laboratories and is considered as an alternative to microscopy [20] particularly in latent infections. In our study as well, PCR was able to identify more than twice the number of cases as detected by microscopy.

There were no uncharacteristic clinical signs in the animals found positive for haemoprotozoan and rickettsial infection. High temperature, pale mucous membranes and decrease in milk production were however prominent signs. The endogenous pyrogens released in the blood due to cellular lysis lead to fever and consequently, inappetence [17]. Anemia occurs due to erythrophagocytosis, lysis of RBCs due to parasite multiplication and subsequent removal by reticuloendothelial system [21, 22]. Consequently, the vital blood parameters which include level of haemoglobin, PCV, MCHC, MCV, TLC and TEC get deranged and further aggravated by the continuous loss of blood sucked out by the ticks [23]. The impact of haemoprotozoan and rickettsial infection on the milk production is a major economical set back. Our results reflect the deviations in the blood parameters following the same cascade and are in agreement with [15, 17, 24].

The study suggests that outbreaks are likely to occur in the rainy and post rainy season due to interplay of epidemiological factors. During rainy season the epizootiological determinants such as ambient temperature and atmospheric humidity and microclimate of grazing lands are favourable for growth and development of ticks. This is unlikely to differ in other parts of country as well, based on earlier reports viz. 29.31% [25] and 58.55% [26]. The vector population was observed as a major differential factor in the prevalence of TBDs in organised and unorganized farms (*p* = 0.007), latter showing increased prevalence. These farms are Kuchha houses (non cemented) or made of bricks only, having cracks and crevices, with improper drainage and poor ventilation, favouring survival and breeding of vector ticks. Moreover, the owners of unorganized farms are comparatively uneducated, so lack of awareness regarding use of acaricidals and sustainable managemental practices against ticks was observed at the time of sample collection. The other determinants recorded for disease occurrence are age and breed of animal. Inverse age resistance shields the young population from clinical outbreak of diseases [27]. The highest prevalence (37.50%) was observed in animals > 3 years of age which is supported by studies from [26, 28]. Also, the adult cattle are predisposed to various stresses due to cycling heat, production, vaccination and reproduction which may augment the pathogenesis of diseases. Cross bred animals revealed higher prevalence of infection (33.62%) than indigenous breeds (15.38%), in agreement with earlier reports [26, 29]. Some workers have attributed susceptibility to the difference in the immune response to produce pro-inflammatory cytokine, which is higher in exotic animals or native breeds harbouring genetic loci for greater tolerance [30, 31].

Intracellular haemoprotozoons are under constant pressure from the immune system of their hosts, leading to emergence of genetic variants [32]. The genetic polymorphism of *Babesia* spp. has been reported earlier using sequence information of 18S rRNA gene [1, 33, 34]. In the phylogenetic tree, the isolates of the present study formed a separate sub clade but clustered with an isolate from Iraq. However, these distinguished itself from South African and American isolates, simulating reports from Kerala [1], wherein the isolates appeared in two different clades. In another study from India [34], close genetic relatedness was observed between *B. bigemina* isolates from North Eastern India with Argentina and Kenya rather than with China. Sequence information of *A. marginale* isolates from the present study revealed a marked divergence from *A. capra, A. central* and *A. ovis*, although, small length of nucleotide sequence couldn’t reveal any marked genetic heterogeneity from isolates of *A. marginale* across the world. In a study from south India, minimal heterogeneity was revealed within 16S rRNA and *msp4* genes among the field isolates from Kerala [1].

The key mechanism responsible for genetic diversity among *Theileria* spp., is recombination during sexual reproduction [35]. The Tams1 gene has been shown to be a promising candidate for carrying antigenic diversity studies in *T. annulata* parasites [36, 37], however some studies have suggested no geographic specificity and other showing region specificity based on the gene polymorphism [36–38]. In the present study, *T. annulata* showed 99% nucleotide homology with Indian isolates and clustered together with an Indian isolate in the phylogenetic clade. Parasite diversity reports from India suggests that the Indian isolates were distributed into two groups along with other countries like Spain, Italy, Tunisia, Iran, Bahrain, Turkey and Iraq [39, 40]. The genetic diversity among the parasite strains can be one of the reasons for vaccination failures and inability to constraint the disease.

## Conclusions

In conclusion the study provided a holistic picture about the prevalence of haemoprotozoon and rickettsial infection in cattle and its correlation with the environmental variables and epidemiological determinants. In addition, the hematological data may be presumed as a marker of health status of animal and its implications on the productivity of animal. The present study is probably the first report of molecular characterization of the haemoprotozoon and rickettsial infection from Jammu region of North India. It provides pioneering information about the circulating genotypes and possible diversities which are quintessential for developing vaccine and diagnostic strategies in future.

## Methods

### Study area and sample collection strategy

The study was conducted in Jammu province of Union Territory of Jammu and Kashmir, India, located at 32.73 ^o^N 74.87 °E. To collect maximum number of samples, the expected prevalence of 20% with confidence limits of 95% and a desired absolute precision of 5% was considered. The number of samples thus calculated was adjusted for finite population [9] and correlated with 278 samples between October, 2017 and September 2019. At the time of blood sample collection, data related to clinical signs, age, sex, breed and type of farm were also recorded. The dairy animals were examined thoroughly and observed for pyrexia, pale mucous membrane, respiratory distress, diarrhea, tick infestation, haemoglobinurea, lymph node enlargement and nasal discharges. Also, the history of anorexia and decrease in milk production was confirmed from the animal owners. The housing system (organized or unorganized) was also taken into consideration as a variable for disease prevalence taking into account the type of flooring, drainage and ventilation of the shed. Blood was collected from the jugular vein into EDTA-coated vacutainer tubes, transported to the laboratory on ice. Thin blood smears were made, fixed in methanol, air dried and stained with Giemsa stain (1:20) as per standard protocol. Stained smears were examined microscopically under oil immersion and approximately 20–35 fields were examined. Blood was stored at − 20 °C until further analysis.

### Disease prevalence and correlation with environmental variables

According to Indian meteorological department Pune, Ministry of earth sciences, Government of India, seasonal variability in the region for the year can be classified into summer (March to June), monsoon/rainy (July to September), postmonsoon/post rains (October to November) and winter (December to February). Hence study period was divided as per climatic variables and correlations were drawn with environmental variables like temperature, relative humidity and rainfall. Metrological data was obtained from the metrology observatory installed at Division of Agrometeorology, SK University of agricultural sciences and technology of Jammu.

### Haematological parameters

The various blood parameters of clinically suspected blood samples were measured with automatic blood analyser (Mythic™ 18 Vet, Orphee, Switzerland). The various attributes included white blood cell count (WBC) (× 10^3^/μl), lymphocytes (%), monocytes (%), granulocytes (%), red blood cell count (RBC) (10^6^/mm^3^), haemoglobin (Hb) (g/dl), platelet count (× 10^3^/μl), mean corpuscular haemoglobin (MCH) (picogram), mean corpuscular haemoglobin concentration (MCHC) (g/dl), mean corpuscular volume (MCV) (femtolitre) and packed cell volume (PCV) (%).

### Genomic DNA extraction and polymerase chain reaction

Genomic DNA was extracted from 200 μl of the whole blood using DNA extraction kit (DNeasy blood kit, Qiagen) following the manufacturer’s protocols. Concentration of isolated DNA was measured using spectrophotometer (Eppendorf, India) and purity was checked in agarose gel electrophoresis (1.5% gel). Aliquots of extracted DNA were stored at − 20 °C until further use.

Each blood sample was subjected to three PCR reactions for detection of *Babesia* spp., *A. marginale* and *T. annulata* targeting 18S rRNA, 16S rRNA and Tams1 gene, respectively [10] with some minor modifications. The oligonucleotide primers used are as follows:

**Table:** 

**Organism**	**Primers**
*Babesia* spp.	Bb 18S F 5 ^′^ -TCCATTCAAGTTTCTGCCCCCATCA- 3^′^
	Bb 18S R 5 ^′^-CCATTACCAAGGCTCAAAAGCAACAA**-** 3^′^
*A. marginale*	Amar 16SF 5^′^ - GGCGGTGATCTGTAGCTGGTCTGA- 3^′^
	Amar 16SR 5 ^′-^ GCCCAATAATTCCGAACAACGCTT- 3^′^
*T. annulata*	Tamulti-F 5^′^ -CCGTTAATGCTGCAAATGAGGAGG- 3^′^
	Tamulti-R 5^′^ -GAGGCGAAGACTGCAAGGGGAG- 3^′^

The PCR reaction was carried out in 25 μl total volume containing 4 μl of template DNA, 2.5 μl of 10× PCR Green buffer (Thermo Scientific, USA), 0.5 μl of 10 mM dNTP, 0.5 μl of each forward and reverse primer (20 pmol/μl), 0.2 μl Dream Taq DNA polymerase and nuclease free water to make the volume 25 μl. Amplification was performed using a S1000 thermal cycler (Bio-Rad, USA) under following conditions: For *Babesia* spp. initial denaturation at 94 °C for 5 min, followed by 35 amplification cycles (94 °C for 1 min, 57 °C for 1 min and 72 °C for 1 min). For *A. marginale,* initial denaturation at 94 °C for 5 min, followed by 35 amplification cycles (94 °C for 45 s, 58 °C for 45 s and 72 °C for 45 s). For *T. annulata*, initial denaturation at 95 °C for 5 min, followed by 37 amplification cycles (95 °C for 30s, 55 °C for 30s and 72 °C for 30s). A final extension step at 72 °C for 15 min was followed after amplification cycles in all three reactions. Known positive genomic DNA of *Babesia* spp., *A. marginale* and *T. annulata* was used as positive control, while nuclease free water was used as negative control. The PCR products were electrophoresed in 1.5% agarose gel (Tris-borate-EDTA), incorporated with ethidium bromide (0.5 μg/ml) and visualized under transilluminator (Eppendorf, India).

### Sequencing and phylogenetic analysis

PCR amplified product of each selected isolate was gel purified using PCR clean up system (Promega, USA). The eluted product was commercially sequenced in an automated DNA sequencer at Agrigenome Pvt. Ltd., Kochi, Kerala. Nucleotide sequences (*n* = 6) generated in the study were primarily analysed using BioEdit software and submitted to GenBank. The sequences were compared with the available sequences in GenBank using BLAST program of NCBI. The sequences were aligned with clustal W programme of MEGA 6 software using gap opening penalty of 10 and gap extension penalty of 0.1 and 0.2 for the pair wise and multiple alignments, respectively. Phylogenetic trees were constructed using maximum parsimony (MP) with the tree–bisection–regrafting (TBR) algorithm and tested at 1000 bootstrap replications. The sequences were initially truncated at both ends, so as to obtain sequences that started and ended at the homologous nucleotide positions. *Plasmodium falciparum* (JQ627149), *Rickettsia rickettsii* (U11021.1) and *Theileria lestoquardi* (KY965146.1) sequences were used as outgroup members to root the respective trees.

### Statistical analysis

Chi-square test and univariate logistic regression models were used to draw inferences from risk factors associated with prevalence of haemoparasitic infections. Haematological alterations were analyzed by one-way analysis of variance (ANOVA) using SPSS 16 software [11].

## Supplementary Information

**Additional file 1.**

**Additional file 2.**

**Additional file 3.**

## Abbreviations

TTBD: Tick and tick borne diseases
RNA: Ribonucleic acid DNA: deoxyribonucleic acid
PCR: Polymerase chain reaction
Hb: Haemoglobin
PCV: Packed cell volume
RBC: Red blood cells
WBC: White blood cells
LYM: Lymphocytes
MON: Monocytes
GRA: Granulocytes
MCH: Mean corpuscular haemoglobin
MCHC: Mean corpuscular haemoglobin concentration
MCV: Mean corpuscular volume.
