# Supplementary material for: Epidemiology, haematology and molecular characterization of haemoprotozoon and rickettsial organisms causing infections in cattle of Jammu region, North India
Source: BMC Vet Res. 2021 Jun 15;17:219. doi: 10.1186/s12917-021-02915-9 (PMC8207732; doi:10.1186/s12917-021-02915-9)
Supplement: Supplementary file 2 — Additional file 2. [file 12917_2021_2915_MOESM2_ESM.pdf]

T C C A T T C A A G T T T C T G C C C C A T C A G Majority
10 20
1 T C C A T T C A A G T T T C T G C C C C A T C A G B.bigemina_Jammu_MN566925.1
1 T C C A T T C A A G T T T C T G C C C C A T C A G B.bigemina_Kathua_MN567603
1 T C C A T T C A A G T T T C T G C C C C A T C A G B.bigemina_udhampur_MN566924.1

C T T G A C G G T A G G G T A T T G G C C T A C C Majority
30 40 50
26 C T T G A C G G T A G G G T A T T G G C C T A C C B.bigemina_Jammu_MN566925.1
26 C T T G A C G G T A G G G T A T T G G C C T A C C B.bigemina_Kathua_MN567603
26 C T T G A C G G T A G G G T A T T G G C C T A C C B.bigemina_udhampur_MN566924.1

G A G G C A G C A A C G G G T A A C G G G G A A T Majority
60 70
51 G A G G C A G C A A C G G G T A A C G G G G A A T B.bigemina_Jammu_MN566925.1
51 G A G G C A G C A A C G G G T A A C G G G G A A T B.bigemina_Kathua_MN567603
51 G A G G C A G C A A C G G G T A A C G G G G A A T B.bigemina_udhampur_MN566924.1

T A G G G T T C G A T T C C G G A G A G G G A G C Majority
80 90 100
76 T A G G G T T C G A T T C C G G A G A G G G A G C B.bigemina_Jammu_MN566925.1
76 T A G G G T T C G A T T C C G G A G A G G G A G C B.bigemina_Kathua_MN567603
76 T A G G G T T C G A T T C C G G A G A G G G A G C B.bigemina_udhampur_MN566924.1

C T G A G A A A C G G C T A C C A C A T C T A A G Majority
110 120
101 C T G A G A A A C G G C T A C C A C A T C T A A G B.bigemina_Jammu_MN566925.1
101 C T G A G A A A C G G C T A C C A C A T C T A A G B.bigemina_Kathua_MN567603
101 C T G A G A A A C G G C T A C C A C A T C T A A G B.bigemina_udhampur_MN566924.1

G A A G G C A G C A G G C G C G C A A A T T A C C Majority
130 140 150
126 G A A G G C A G C A G G C G C G C A A A T T A C C B.bigemina_Jammu_MN566925.1
126 G A A G G C A G C A G G C G C G C A A A T T A C C B.bigemina_Kathua_MN567603
126 G A A G G C A G C A G G C G C G C A A A T T A C C B.bigemina_udhampur_MN566924.1

C A A T C C T G A C A C A G G G A G G T A G T G A Majority
160 170
151 C A A T C C T G A C A C A G G G A G G T A G T G A B.bigemina_Jammu_MN566925.1
151 C A A T C C T G A C A C A G G G A G G T A G T G A B.bigemina_Kathua_MN567603
151 C A A T C C T G A C A C A G G G A G G T A G T G A B.bigemina_udhampur_MN566924.1

C A A G A A A T A A C A A T A C A G G G C T T T C Majority
180 190 200
176 C A A G A A A T A A C A A T A C A G G G C T T T C B.bigemina_Jammu_MN566925.1
176 C A A G A A A T A A C A A T A C A G G G C T T T C B.bigemina_Kathua_MN567603
176 C A A G A A A T A A C A A T A C A G G G C T T T C B.bigemina_udhampur_MN566924.1

G T C T T G T A A T T G G A A T G A T G G T G A T Majority
210 220
201 G T C T T G T A A T T G G A A T G A T G G T G A T B.bigemina_Jammu_MN566925.1
201 G T C T T G T A A T T G G A A T G A T G G T G A T B.bigemina_Kathua_MN567603
201 G T C T T G T A A T T G G A A T G A T G G T G A T B.bigemina_udhampur_MN566924.1

Start	Sequence	Name
	G T A C A A C C T C A C C A G A G T A C C A A T T	Majority
226	G T A C A A C C T C A C C A G A G T A C C A A T T	B.bigemina_Jammu_MN566925.1
226	G T A C A A C C T C A C C A G A G T A C C A A T T	B.bigemina_Kathua_MN567603
226	G T A C A A C C T C A C C A G A G T A C C A A T T	B.bigemina_udhampur_MN566924.1

Start	Sequence	Name
	G G A G G G C A A G T C T G G T G C C A G C A G C	Majority
251	G G A G G G C A A G T C T G G T G C C A G C A G C	B.bigemina_Jammu_MN566925.1
251	G G A G G G C A A G T C T G G T G C C A G C A G C	B.bigemina_Kathua_MN567603
251	G G A G G G C A A G T C T G G T G C C A G C A G C	B.bigemina_udhampur_MN566924.1

Start	Sequence	Name
	C G C G G T A A T T C C A G C T C C A A T A G C G	Majority
276	C G C G G T A A T T C C A G C T C C A A T A G C G	B.bigemina_Jammu_MN566925.1
276	C G C G G T A A T T C C A G C T C C A A T A G C G	B.bigemina_Kathua_MN567603
276	C G C G G T A A T T C C A G C T C C A A T A G C G	B.bigemina_udhampur_MN566924.1

Start	Sequence	Name
	T A T A T T A A A C T T G T T G C A G T T A A A A	Majority
301	T A T A T T A A A C T T G T T G C A G T T A A A A	B.bigemina_Jammu_MN566925.1
301	T A T A T T A A A C T T G T T G C A G T T A A A A	B.bigemina_Kathua_MN567603
301	T A T A T T A A A C T T G T T G C A G T T A A A A	B.bigemina_udhampur_MN566924.1

Start	Sequence	Name
	A G C T C G T A G T T G T A T T T C A G C C T C G	Majority
326	A G C T C G T A G T T G T A T T T C A G C C T C G	B.bigemina_Jammu_MN566925.1
326	A G C T C G T A G T T G T A T T T C A G C C T C G	B.bigemina_Kathua_MN567603
326	A G C T C G T A G T T G T A T T T C A G C C T C G	B.bigemina_udhampur_MN566924.1

Start	Sequence	Name
	C G T T T T T T C C C T G G T T T T G G G T C T T	Majority
351	C G T T T T T T C C C T C G T T T T G G G T C T T	B.bigemina_Jammu_MN566925.1
351	C G T T T T T T C C C T G G T T T T G G G T C T T	B.bigemina_Kathua_MN567603
351	C G T T T T T T C C C T G G T T T T G G G T C T T	B.bigemina_udhampur_MN566924.1

Start	Sequence	Name
	T T C G C T G G C T T T T T T T T T A C T T T G A	Majority
376	T T C G C T G G C T T T T T T T T T A C T T T G A	B.bigemina_Jammu_MN566925.1
376	T T C G C T G G C T T T T T T T T T A C T T T G A	B.bigemina_Kathua_MN567603
376	T T C G C T G G C T T T T T T T T T A C T T T G A	B.bigemina_udhampur_MN566924.1

Start	Sequence	Name
	G A A A A T T A G A G T G T T T C A A G C A G A C	Majority
401	G A A A A T T A G A G T G T T T C A A G C A G A C	B.bigemina_Jammu_MN566925.1
401	G A A A A T T A G A G T G T T T C A A G C A G A C	B.bigemina_Kathua_MN567603
401	G A A A A T T A G A G T G T T T C A A G C A G A C	B.bigemina_udhampur_MN566924.1

Start	Sequence	Name
	T T T T G T C T T G A A T A C T T C A G C A T G G	Majority
426	T T T T G T C T T G A A T A C T T C A G C A T G G	B.bigemina_Jammu_MN566925.1
426	T T T T G T C T T G A A T A C T T C A G C A T G G	B.bigemina_Kathua_MN567603
426	T T T T G T C T T G A A T A C T T C A G C A T G G	B.bigemina_udhampur_MN566924.1

```
     A A T A A T A G A G T A G G A C C T T G G T T C T   Majority
                       460                 470
451  A A T A A T A G A G T A G G A C C T T G G T T C T   B.bigemina_Jammu_MN566925.1
451  A A T A A T A G A G T A G G A C C T T G G T T C T   B.bigemina_Kathua_MN567603
451  A A T A A T A G A G T A G G A C C T T G G T T C T   B.bigemina_udhampur_MN566924.1

     A T T T T G T T G C T T T T G A G C C T T G G T A   Majority
             480                 490                 500
476  A T T T T G T T G C T T T T G A G C C T T G G T A   B.bigemina_Jammu_MN566925.1
476  A T T T T G T T G C T T T T G A G C C T T G G T A   B.bigemina_Kathua_MN567603
476  A T T T T G T T G C T T T T G A G C C T T G G T A   B.bigemina_udhampur_MN566924.1

     A T G G                                             Majority

501  A T G G                                             B.bigemina_Jammu_MN566925.1
501  A T G G                                             B.bigemina_Kathua_MN567603
501  A T G G                                             B.bigemina_udhampur_MN566924.1
```

Decoration 'Decoration #1': Shade (with solid deep red) residues that differ from the Consensus.
