# Supplementary material for: Epidemiology, haematology and molecular characterization of haemoprotozoon and rickettsial organisms causing infections in cattle of Jammu region, North India
Source: BMC Vet Res. 2021 Jun 15;17:219. doi: 10.1186/s12917-021-02915-9 (PMC8207732; doi:10.1186/s12917-021-02915-9)
Supplement: Supplementary file 3 — Additional file 3. [file 12917_2021_2915_MOESM3_ESM.pdf]

```
     - - - - - - - - - - - - - - - - - - - - - - - - -   Majority
                       10                  20
1    - - - - - - - - - - - - - - - - - - - - - - - - -   T.annulata_Jammu_MT113479
1    A T G T T G T C C A G G A C C A C C C T C A A G T   T.annulata AF214840.1 Hisar.seq
1    - - - - - - - - - - - - - - - - - - - - - - - - -   T.annulata MF346013.1 Izatnagar.seq

     - - - - - - - - - - - - - - - - - - - - - - - - -   Majority
               30                  40                  50
1    - - - - - - - - - - - - - - - - - - - - - - - - -   T.annulata_Jammu_MT113479
26   T C T T A T A T T T G A G C T T C T T C G T T A T   T.annulata AF214840.1 Hisar.seq
1    - - - - - - - - - - - - - - - - - - - - - - - - -   T.annulata MF346013.1 Izatnagar.seq

     - - - A T C C G T T A A T G C T G C A A A T G A G   Majority
                       60                  70
1    - - - - - C C G T T A A T G C T G C A A A T G A G   T.annulata_Jammu_MT113479
51   C T C A T C C G T T A A T G C T G C A A A T G A G   T.annulata AF214840.1 Hisar.seq
1    - - - A T C C G T T A A T G C T G C A A A T G A G   T.annulata MF346013.1 Izatnagar.seq

     G A G G A A A A G A A A A A G G A G G A A A A A A   Majority
               80                  90                  100
21   G A G G A A A A G A A A A A G G A G G A A A A A A   T.annulata_Jammu_MT113479
76   G A G G A A A A G A A A A A G G A G G A A A A A A   T.annulata AF214840.1 Hisar.seq
23   G A G G A A A A G A A A A A G G A G G A A A A A A   T.annulata MF346013.1 Izatnagar.seq

     A A G A T G T T G T T C T C G A T G T T A C T C T   Majority
                       110                 120
46   A A G A T G T T G T T C T C G A T G T T A C T C T   T.annulata_Jammu_MT113479
101  A A G A T G T T G T T C T C G A T G T T A C T C T   T.annulata AF214840.1 Hisar.seq
48   A A G A T G T T G T T C T C G A T G T T A C T C T   T.annulata MF346013.1 Izatnagar.seq

     C A C T T C A T G T G A G A A T G T A A C C T T T   Majority
               130                 140                 150
71   C A C T T C A T G T G A G A A T G T A A C C T T T   T.annulata_Jammu_MT113479
126  C A C T T C A T G T G A G A A T G T A A C C T T T   T.annulata AF214840.1 Hisar.seq
73   C A C T T C A T G T G A G A A T G T A A C C T T T   T.annulata MF346013.1 Izatnagar.seq

     A A A A A C G T C G A C C C T A A C A C C A C T G   Majority
                       160                 170
96   A A A A A C G T C G A C C C T A A C A C C A C T G   T.annulata_Jammu_MT113479
151  A A A A A C G T C G A C C C T A A C A C C A C T G   T.annulata AF214840.1 Hisar.seq
98   A A A A A C G T C G A C C C T A A C A C C A C T G   T.annulata MF346013.1 Izatnagar.seq

     A G T T A A C T G T C A A A G A C G G C T T C C G   Majority
               180                 190                 200
121  A G T T A A C T G T C A A A G A C G G C T T C C G   T.annulata_Jammu_MT113479
176  A G T T A A C T G T C A A A G A C G G C T T C C G   T.annulata AF214840.1 Hisar.seq
123  A G T T A A C T G T C A A A G A A G G C T T C C G   T.annulata MF346013.1 Izatnagar.seq

     T T T C A A G A C C C T T A A G G T C G G A G A C   Majority
                       210                 220
146  T T T C A A G A C C C T T A A G G T C G G A G A C   T.annulata_Jammu_MT113479
201  T T T C A A G A C C C T T A A G G T C G G A G A C   T.annulata AF214840.1 Hisar.seq
148  T T T C A A G A C C C T T A A G G T C G G A G A C   T.annulata MF346013.1 Izatnagar.seq
```

```
    A A G A C C T T G T T C A A T G T T G A C A C C T   Majority
            230                 240                 250
171 A A G A C C T T G T T C A A T G T T G A C A C C T   T.annulata_Jammu_MT113479
226 A A G A C C T T G T T C A A T G T T G A C A C C T   T.annulata AF214840.1 Hisar.seq
173 A A G A C C T T G T T C A A T G T T G A C A C C T   T.annulata MF346013.1 Izatnagar.seq

    C A A A A C A T A C C C C A G T A C A G G C A T T   Majority
                      260                 270
196 C A A A A C A T A C C C C A G T A C A G G C A T T   T.annulata_Jammu_MT113479
251 C A A A A C A T A C C C C A G T A C A G G C A T T   T.annulata AF214840.1 Hisar.seq
198 C A A A A C A T A C C C C A G T A C A G G C A T T   T.annulata MF346013.1 Izatnagar.seq

    C A A A C T T A A G C A T G A A T C C G A T G A G   Majority
            280                 290                 300
221 C A A A C T T A A G C A T G A A T C C G A T G A G   T.annulata_Jammu_MT113479
276 C A A A C T T A A G C A T G A A T C C G A T G A G   T.annulata AF214840.1 Hisar.seq
223 C A A A C T T A A G C A T G A A T C C G A T G A G   T.annulata MF346013.1 Izatnagar.seq

    T G G T T C A G A C T T A A T C T T C A T G C T G   Majority
                      310                 320
246 T G G T T C A G A C T T A A T C T T C A T G C T G   T.annulata_Jammu_MT113479
301 T G G T T C A G A C T T A A T C T T C A T G C T G   T.annulata AF214840.1 Hisar.seq
248 T G G T T C A G A C T T A A T C T T C A T G C T G   T.annulata MF346013.1 Izatnagar.seq

    C C C A G C C A A A G A T G T T C A A G A A G A A   Majority
            330                 340                 350
271 C C C A G C C A A A G A T G T T C A A G A A G A A   T.annulata_Jammu_MT113479
326 C C C A G C C A A A G A T G T T C A A G A A G A A   T.annulata AF214840.1 Hisar.seq
273 C C C A G C C A A A G A T G T T C A A G A A G A A   T.annulata MF346013.1 Izatnagar.seq

    G G G A G A C A A G G A A T A T T C T G A G G T C   Majority
                      360                 370
296 G G G A G A C A A G G A A T A T T C T G A G G T C   T.annulata_Jammu_MT113479
351 G G G A G A C A A G G A A T A T T C T G A G G T C   T.annulata AF214840.1 Hisar.seq
298 G G G A G A C A A G G A A T A T T C T G A G G T C   T.annulata MF346013.1 Izatnagar.seq

    A A A T T C G A G A C C T A C T A C G A T G A T G   Majority
            380                 390                 400
321 A A A T T C G A G A C C T A C T A C G A T G A T G   T.annulata_Jammu_MT113479
376 A A A T T C G A G A C C T A C T A C G A T G A T G   T.annulata AF214840.1 Hisar.seq
323 A A A T T C G A G A C C T A C T A C G A T G A T G   T.annulata MF346013.1 Izatnagar.seq

    T C T T G T T C A A G G G A A A A T C C G C C A A   Majority
                      410                 420
346 T C T T G T T C A A G G G A A A A T C C G C C A A   T.annulata_Jammu_MT113479
401 T C T T G T T C A A G G G A A A A T C A G C A A A   T.annulata AF214840.1 Hisar.seq
348 T C T T G T T C A A G G G A A A A T C C G C C A A   T.annulata MF346013.1 Izatnagar.seq

    G G A A C T A G A T G T T T C C A A A T T C G A A   Majority
            430                 440                 450
371 G G A A C T A G A T G T T T C C A A A T T C G A A   T.annulata_Jammu_MT113479
426 G G A A C T A G A T G T T T C C A A G T T C G A A   T.annulata AF214840.1 Hisar.seq
373 G G A A C T A G A T G T T T C C A A A T T C G A A   T.annulata MF346013.1 Izatnagar.seq
```

Position	Sequence	Name
	G A T A C A G C T T T G T T C A C C G C C T C C G	Majority
	460 470	
396	G A T A C A G C T T T G T T C A C C G C C T C C G	T.annulata_Jammu_MT113479
451	G A T C C A G C T T T G T T C A C C C C C T C C G	T.annulata AF214840.1 Hisar.seq
398	G A T A C A G C T T T G T T C A C C G C C T C C G	T.annulata MF346013.1 Izatnagar.seq

Position	Sequence	Name
	C C T T T G G C A C T G G A A G G A A G T A C A C	Majority
	480 490 500	
421	C C T T T G G C A C T G G A A G G A A G T A C A C	T.annulata_Jammu_MT113479
476	C C T T C G G C A C T G G A A G G A A G T A C A C	T.annulata AF214840.1 Hisar.seq
423	C C T T T G G C A C T G G A A G G A A G T A C A C	T.annulata MF346013.1 Izatnagar.seq

Position	Sequence	Name
	C T T T A A A A A G G A T T T C A A A C C T T C C	Majority
	510 520	
446	C T T T A A A A A G G A T T T C A A A C C T T C C	T.annulata_Jammu_MT113479
501	C T T T A A A A A G G A T T T C A A A C C T T C C	T.annulata AF214840.1 Hisar.seq
448	C T T T A A A A A G G A T T T C A A A C C T T C C	T.annulata MF346013.1 Izatnagar.seq

Position	Sequence	Name
	A A A G T T C T C T T C G A A A A G A A A G A A G	Majority
	530 540 550	
471	A A A G T T C T C T T C G A A A A G A A A G A A G	T.annulata_Jammu_MT113479
526	A A A G T T C T C T T C G A A A A G A A A G A A G	T.annulata AF214840.1 Hisar.seq
473	A A A G T T C T C T T C G A A A A G A A A G A A G	T.annulata MF346013.1 Izatnagar.seq

Position	Sequence	Name
	T C G G A A A A C C A A A C A A T G C C A A G T A	Majority
	560 570	
496	T C G G A A A A C C A A A C A A T G C C A A G T A	T.annulata_Jammu_MT113479
551	T C G G A A A A C C A A A C A A T G C C A A G T A	T.annulata AF214840.1 Hisar.seq
498	T C G G A A A A C C A A A C A A T G C C A A G T A	T.annulata MF346013.1 Izatnagar.seq

Position	Sequence	Name
	T C T T G A T G T T T T C G T C T T T G T C A G T	Majority
	580 590 600	
521	T C T T G A T G T T T T C G T C T T T G T C A G T	T.annulata_Jammu_MT113479
576	T C T T G A T G T T T T C G T C T T T G T C A G T	T.annulata AF214840.1 Hisar.seq
523	T C T T G A T G T T T T C G T C T T T G T C A G T	T.annulata MF346013.1 Izatnagar.seq

Position	Sequence	Name
	G C T G A T T C C A A G A A G G T C G T C A G A C	Majority
	610 620	
546	G C T G A T T C C A A G A A G G T C G T C A G A C	T.annulata_Jammu_MT113479
601	G C T G A T T C C A A G A A G G T C G T C A G A C	T.annulata AF214840.1 Hisar.seq
548	G C T G A T T C C A A G A A G G T C G T C A G A C	T.annulata MF346013.1 Izatnagar.seq

Position	Sequence	Name
	T C G A C T A C T T C T A T A C C G G T G A C T C	Majority
	630 640 650	
571	T C G A C T A C T T C T A T A C C G G T G A C T C	T.annulata_Jammu_MT113479
626	T C G A C T A C T T C T A T A C C G G T G A C T C	T.annulata AF214840.1 Hisar.seq
573	T C G A C T A C T T C T A T A C C G G T G A C T C	T.annulata MF346013.1 Izatnagar.seq

Position	Sequence	Name
	A A G G T T G A A G G A G A C C T A C T T C G A G	Majority
	660 670	
596	A A G G T T G A A G G A G A C C T A C T T C G A G	T.annulata_Jammu_MT113479
651	A A G G T T G A A G G A G A C C T A C T T C G A G	T.annulata AF214840.1 Hisar.seq
598	A A G G T T G A A G G G G A C A T A C T T C G A G	T.annulata MF346013.1 Izatnagar.seq

	C T T A A G G A C G A T A A G T G G G T A C A A A	Majority
	680 690 700	
621	C T T A A G G A C G A T A A G T G G G T A C A A A	T.annulata_Jammu_MT113479
676	C T T A A G G A C G A T A A G T G G G T A C A A A	T.annulata AF214840.1 Hisar.seq
623	C T T A A G G A C G A T A A G T G G G T A C A A A	T.annulata MF346013.1 Izatnagar.seq

	T G T C A C A G G C A G A T G C A A A C A A G G C	Majority
	710 720	
646	T G T C A C A G G C A G A T G C A A A C A A G G C	T.annulata_Jammu_MT113479
701	T G T C A C A G G C A G A T G C A A A C A A G G C	T.annulata AF214840.1 Hisar.seq
648	T G T C A C A G G C A G A T G C A A A C A A G G C	T.annulata MF346013.1 Izatnagar.seq

	C T T G A A C G C C A T G G A C T C A T C A T G G	Majority
	730 740 750	
671	C T T G A A C G C C A T G G A C T C A T C A T G G	T.annulata_Jammu_MT113479
726	C T T G A A C G C C A T G G A C T C A T C A T G G	T.annulata AF214840.1 Hisar.seq
673	C T T G A A C G C C A T G G A C T C A T C A T G G	T.annulata MF346013.1 Izatnagar.seq

	T C A T C C G A T T A C A A A C C A G T T G T C G	Majority
	760 770	
696	T C A T C C G A T T A C A A A C C A G T T G T C G	T.annulata_Jammu_MT113479
751	T C A T C C G A T T A C A A A C C A G T T G T C G	T.annulata AF214840.1 Hisar.seq
698	T C A T C C G A T T A C A A A C C A G T T G T C G	T.annulata MF346013.1 Izatnagar.seq

	A C A A G T T C T C T C C C C T T G C A G T C T T	Majority
	780 790 800	
721	A C A A G T T C T C T C C C C T T G C A G T C T T	T.annulata_Jammu_MT113479
776	A C A A G T T C T C T C C C C T T G C A G T C T T	T.annulata AF214840.1 Hisar.seq
723	A C A A G T T C T C T C C C C T T G C A G T C T T	T.annulata MF346013.1 Izatnagar.seq

	C G C C T C A X X X X X X X X X X X X X X X X X X	Majority
	810 820	
746	C G C C T C	T.annulata_Jammu_MT113479
801	C G C C T C A G T A C T C A T C G T C T T C T C A	T.annulata AF214840.1 Hisar.seq
748	C G C C T C A	T.annulata MF346013.1 Izatnagar.seq

	X X X X X X X X X X X X X X X X X X X X X	Majority
	830 840	
751		T.annulata_Jammu_MT113479
826	T C A G T C C T T T A C T T C C T T T A A	T.annulata AF214840.1 Hisar.seq
754		T.annulata MF346013.1 Izatnagar.seq

Decoration 'Decoration #1': Shade (with solid bright yellow) residues that differ from the Consensus.
